# Patients with pathological stage N2 rectal cancer treated with early adjuvant chemotherapy have a lower treatment failure rate

**DOI:** 10.1186/s12885-017-3170-3

**Published:** 2017-03-09

**Authors:** Yan-Ru Feng, Jing Jin, Hua Ren, Xin Wang, Shu-Lian Wang, Wei-Hu Wang, Yong-Wen Song, Yue-Ping Liu, Yuan Tang, Ning Li, Xin-Fan Liu, Hui Fang, Zi-Hao Yu, Ye-Xiong Li

**Affiliations:** Department of Radiation Oncology, National Cancer Center/Cancer Hospital, Chinese Academy of Medical Sciences, Peking Union Medical College, Beijing, 100021 China

**Keywords:** Adjuvant chemoradiotherapy, Adjuvant chemotherapy, Sequence, Rectal cancer

## Abstract

**Background:**

In this era of oxaliplatin-based adjuvant therapy, the optimal sequence in which chemoradiotherapy should be administered for pathological stage N2 rectal cancer is unknown. The aim of this study was to investigate this sequence.

**Methods:**

In the primary adjuvant concurrent chemoradiotherapy (A-CRT) group (*n* = 71), postoperative concurrent chemoradiotherapy was administered before adjuvant chemotherapy. In the primary adjuvant chemotherapy (A-CT) group (*n* = 43), postoperative concurrent chemoradiotherapy was administered during or after adjuvant chemotherapy. Postoperative radiotherapy comprised 45–50.4 Gy in 25–28 fractions. Concurrent chemotherapy comprised two cycles of oral capecitabine (1,600 mg/m^2^) on days 1–14 and 22–35. Patients receiving adjuvant chemotherapy with four or more cycles of XELOX (oxaliplatin plus capecitabine) or eight or more cycles of FOLFOX (fluorouracil, leucovorin, and oxaliplatin) were included.

**Results:**

Between June 2005 and December 2013, data for 114 qualified rectal cancer patients were analyzed. The percentages of patients in whom treatment failed in the A-CRT and A-CT groups were 33.8% and 16.3%, respectively (*p* = 0.042). More patients had distant metastases in the A-CRT group than in the A-CT group (32.4% vs. 14.3%, *p* = 0.028). Multivariate analysis indicated that the sequence in which chemoradiotherapy was administered (A-CT vs. A-CRT) was an independent prognostic factor for both estimated disease-free survival [hazard ratio (HR) 0.345, 95% confidence interval (CI) 0.137–0.868, *p* = 0.024] and estimated distant metastasis-free survival (HR 0.366, 95% CI 0.143–0.938, *p* = 0.036).

**Conclusions:**

In pathological stage N2 rectal cancer patients, administering adjuvant chemotherapy before chemoradiotherapy led to a lower rate of treatment failure, especially with respect to distant metastasis. Adjuvant chemotherapy prescribed as early as possible might benefit this cohort of patients in this era of oxaliplatin-based adjuvant therapy.

## Background

Since the pivotal German trial, preoperative chemoradiotherapy following surgery has been preferred for locally advanced rectal cancer in the routine practice of most institutions [1]. However, postoperative chemoradiotherapy and adjuvant chemotherapy are still recommended for patients with pathological stage II/III disease after definitive surgery without preoperative chemoradiotherapy [2].

Among patients with pathological stage N2 rectal cancer treated with curative intent, about 40% will have distant metastases and 24% local recurrence at 5 years [3, 4]. Optimizing the combination of radiotherapy and chemotherapy is therefore necessary to reduce recurrence. Trials investigating patients with stage II/III rectal cancer indicated that the sequence in which chemoradiotherapy was administered was not associated with disease-free survival (DFS), overall survival (OS) or relapse rate [5, 6]. However, there are no reports focusing on pathological stage N2 patients. The ADORE trial and the CAO/ARO/AIO-04 trial indicated the benefit of adjuvant oxaliplatin-based chemotherapy for rectal cancer [7, 8]. In view of the use of leucovorin-modulated fluorouracil chemotherapy and the inclusion of stage II rectal cancer in the previous studies [5, 6], the aim of the present study was to evaluate the sequence in which chemoradiotherapy should be administered for pathological stage N2 rectal cancer in this era of oxaliplatin-based adjuvant therapy.

## Methods

### Patients and patient workup

Treatment outcomes were analyzed for pathological stage N2 rectal cancer patients after curative surgery and the administration of differing sequences of adjuvant concurrent chemoradiotherapy and chemotherapy. The inclusion criteria were as follows: 1) postoperative (R0 resection) pathological stage N2 rectal adenocarcinoma; 2) no evidence of distant metastasis; 3) Karnofsky performance score ≥ 70; 4) receiving postoperative capeciatbine based concurrent chemoradiotherapy; 5) receiving adjuvant chemotherapy [four or more cycles of XELOX (oxaliplatin plus capecitabine) or eight or more cycles of FOLFOX (fluorouracil, leucovorin, and oxaliplatin)]; 6) no neoadjuvant (chemo) radiotherapy; 7) no pregnancy or lactation; and 8) no previous malignancy or other concomitant malignant disease.

In the primary adjuvant concurrent chemoradiotherapy (A-CRT) group, postoperative concurrent chemoradiotherapy was administered before adjuvant chemotherapy. In the primary adjuvant chemotherapy (A-CT) group, postoperative concurrent chemoradiotherapy was administered during or after adjuvant chemotherapy. The pretreatment workup included a complete history and physical examination, liver and renal biochemical analysis, complete blood cell count, electrocardiography, carcino-embryonic antigen determination, abdominal ultrasonography and/or computed tomography (CT), pelvic CT or magnetic resonance imaging (MRI) and chest radiography. All patients underwent disease staging using the American Joint Committee on Cancer 2010 staging system.

### Treatment

Postoperative radiotherapy comprised 45–50.4 Gy (minimum photon energy of 6 MV) in 25–28 fractions of 1.8 or 2.0 Gy five times per week over 5–5.5 weeks. This dose was delivered using three-field conventional radiotherapy, three-dimensional conformal radiotherapy or intensity-modulated radiotherapy technique. The clinical target volume was delineated according to Roels’ guidelines [9], as in previous studies [10–12]. Concurrent chemotherapy comprised two cycles of oral capecitabine (1,600 mg/m^2^) on days 1–14 and 22–35. Perioperative therapy with XELOX, FOLFOX4 or mFOLFOX6 for a total of 6 months is recommended for patients with stage N2 rectal cancer [2].

### Follow-up

Follow-up included physical examination, liver and renal biochemistry, complete blood count, and measurement of tumor markers every 3 months for the first 2 years, and every 6 months thereafter. Abdominal ultrasonography and/or CT, pelvic CT or MRI and chest radiography were performed every 6 months. Colonoscopic examination was repeated annually. Treatment-induced toxicities were scored according to the Common Terminology Criteria for Adverse Events version 3.0.

### Statistical analysis

SPSS version 22.0 (IBM, Armonk, NY, USA) was used for statistical analysis. The OS, DFS, locoregional recurrence-free survival (LRFS), and distant metastasis-free survival (DMFS) were measured from the day of surgery to the date of the event. Survival data were evaluated using the Kaplan–Meier method. The log-rank test was used in univariate analysis to compare survival outcomes between the A-CRT and A-CT groups. Multivariate analysis using a Cox proportional hazards model was used to test independent significance by backward elimination of insignificant explanatory variables. Host factors (age and sex) were included as the covariates in all tests. Chi-square, Fisher exact, and Mann–Whitney *U* tests were used to compare differences between the two groups. Statistical tests were based on a two-sided significance level. *p* < 0.05 indicated statistical significance.

## Results

### Patient characteristics

Between June 2005 and December 2013, data for 114 rectal cancer patients who met all of the inclusion criteria were analyzed retrospectively. Their clinical characteristics are listed in Table 1. There were more patients with stage IIIc disease or tumor deposits in the A-CT group (*p* < 0.05). Radiation dose did not differ between the two groups. However, 93% of patients in the A-CRT group received a full dose of concurrent chemotherapy, compared with 86% of patients in the A-CT group. The median intervals between surgery and the start of adjuvant treatment in the A-CRT and A-CT groups was 6.6 (range 3.6–14.0) weeks and 4.3 (1.9–16.1) weeks, respectively (*p* < 0.001). In the A-CT group, the median number of chemotherapy cycles administered before radiotherapy was four (1–12).

**Table 1:** Clinical characteristics of 114 patients with pathological stage N2 rectal cancer

Characteristics	A-CRT group (*n* = 71)	A-CT group (*n* = 43)	*p*
Sex			0.426
Men	47 (66.2)	26 (60.5)	
Women	24 (33.8)	17 (39.5)	
Age(years)			0. 261
Median	54	52	
Range	32-73	23-70	
Distance from anal verge (cm)			0.773
≤ 5 cm	25 (35.2)	11 (25.6)	
> 5 cm	46 (64.8)	32 (74.4)	
Karnofsky Performance Score			0.464
≥ 90	36 (50.7)	25 (58.1)	
<90	35 (49.3)	18 (41.9)	
pT category			0.062
T2	4 (5.6)	3 (7.0)	
T3	64 (90.1)	32 (74.4)	
T4	3 (4.2)	8 (18.6)	
TNM stage			0.049
IIIB	40 (56.3)	15 (34.9)	
IIIC	31 (43.7)	28 (65.1)	
Surgery			0.438
Low anterior resection	54 (76.1)	35 (81.4)	
Abdominoperineal resection	17 (23.9)	7 (16.3)	
Hartmann	0 (0.0)	1 (2.3)	
Lymphovascular invasion			0.125
Yes	15 (21.1)	13 (30.2)	
No	56 (78.9)	30 (69.8)	
Tumor deposits			0.049
Yes	11 (15.5)	12 (27.9)	
No	60 (84.5)	31 (72.1)	
Number of nodes retrieved			0.080
Median	17	20	
Range	6-51	8-41	
Number of positive nodes			0.050
Median	6	7	
Range	4-29	4-26	
Radiation dose			>0.999
≥ 45Gy	70 (93.6)	43 (100.0)	
<45Gy	1 (1.4)	0 (0.0)	
Concurrent chemotherapy			0.226
Full dose	66 (93.0)	37 (86.0)	
Reduced dose	5 (7.0)	6 (14.0)	
Time to adjuvant treatment (wk)			<0.001
Median	6.6	4.3	
Range	3.6-14.0	1.9-16.1	
Time to adjuvant radiotherapy (wk)			<0.001
Median	6.6	17.1	
Range	3.6-14.0	5.1-35.4	
Time to adjuvant chemotherapy (wk)			<0.001
Median	15.6	4.3	
Range	4.6-23.9	1.9-16.1	

*Abbreviations*: *A-CRT* primary adjuvant concurrent chemoradiotherapy, *A-CT* primary adjuvant chemotherapy

### Failure pattern

For all patients, the median local recurrence time was 26.2 (13.4–59.6) months and the median distant metastasis time was 13.8 (6.5–50.0) months. The percentages of patients in whom treatment failed in the A-CRT and A-CT groups were 33.8% and 16.3%, respectively (*p* = 0.042). More patients had distant metastasis in the A-CRT group than in the A-CT group (32.4% vs. 14.3%, *p* = 0.028). The lung (*n* = 17) was the most common site of distant metastasis, followed by the liver (*n* = 8), the bone (*n* = 4), non-regional lymph nodes (*n* = 4), and the peritoneal seeding (*n* = 3). Details of the patterns of recurrence are shown in Table 2.

**Table 2:** Failure patterns of patients with pathological stage N2 rectal cancer in A-CRT group and A-CT group

Sites of recurrence	A-CRT group		A-CT group		*p*
	*n*	%	*n*	%	
Total no. of recurrence	24	33.8	7	16.3	0.042
No. of locoregional recurrence	4	5.6	1	2.3	0.647
No. of distant metastasis	23	32.4	6	14.3	0.028
All site of distant metastasis					
Lung	14		3		
Liver	7		1		
Bone	3		1		
Non-regional lymph nodes	3		1		
Peritoneal seeding	2		1		

*Abbreviations*: *A-CRT* primary adjuvant concurrent chemoradiotherapy, *A-CT* primary adjuvant chemotherapy

### Survival

The median follow-up time was 34.1 (10.2–112.1) months. For all patients, 3-year estimated OS, DFS, LRFS and DMFS rates were 84.6%, 72.5%, 94.8% and 74.1%, respectively. These rates were 81.8%, 66.7%, 93.7% and 67.5% for patients in the A-CRT group and 90.8%, 83.9%, 97.4% and 86.6% for the A-CT group.

**Fig. 1:**
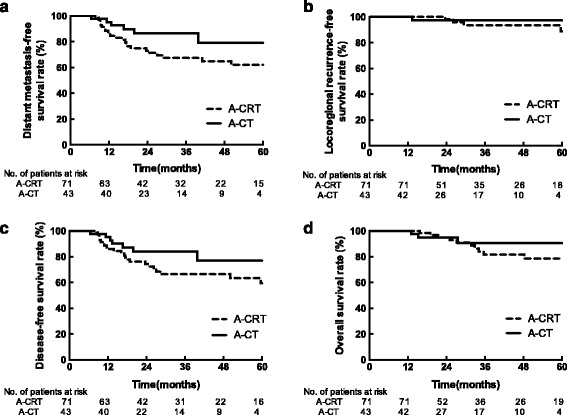
Kaplan–Meier curves of patients with pathological stage N2 rectal cancer treated with primary adjuvant concurrent chemoradiotherapy (A-CRT) or primary adjuvant chemotherapy (A-CT). **a** The 3-year distant metastasis-free survival rates are 67.5% in the A-CRT group and 86.6% in the A-CT group (*p* = 0.074). **b** The 3-year locoregional recurrence-free survival rates are 93.7% in the A-CRT group and 97.4% in the A-CT group (*p* = 0.629). **c** The 3-year disease-free survival rates are 66.7% in the A-CRT group and 83.9% in the A-CT group (*p* = 0.153). **d** The overall survival rates are 81.8% in the A-CRT group and 90.8% in the A-CT group (*p* = 0.378)

Univariate analysis suggested no statistically significant difference in estimated DMFS rate between the A-CRT group and the A-CT group; however, the 3-year estimated DMFS in the A-CRT group was higher (86.6% vs. 67.5%, *p* = 0.074) (Fig. 1a). No statistically significant difference was observed in estimated LRFS, DFS or OS between the A-CRT and the A-CT groups (Fig. 1b, c, d).

Multivariate analysis was performed to adjust for various prognostic factors. The following parameters were included in the Cox proportional hazards model: age, gender, distance from anal verge, lymphovascular invasion, tumor deposits, number of nodes retrieved, number of positive nodes, time to adjuvant treatment, TNM stage and the sequence in which chemoradiotherapy was administered (A-CT vs. A-CRT). The sequence of chemoradiotherapy was identified as an independent prognostic factor for both estimated DFS [hazard ratio (HR) 0.345, 95% confidence interval (CI) 0.137–0.868, *p* = 0.024] and estimated DMFS (HR 0.366, 95% CI 0.143–0.938, *p* = 0.036). The outcomes are shown in Tables 3 and 4.

**Table 3:** Univariate analysis of prognostic factors for 114 patients with stage N2 rectal cancer

Item	n	3y-OS		3y-DFS		3y-LRFS		3y-DMFS	
		%	*P*	%	*P*	%	*P*	%	*P*
Sex									
Men	73	86.0	0.983	75.1	0.831	95.3	0.980	73.9	0.970
Women	41	82.8		68.7		93.9		74.7	
Age(years)									
<60	91	86.5	0.533	71.1	0.280	95.2	0.943	73.0	0.321
≥ 60	23	75.5		75.2		92.3		75.2	
Distance from anal verge (cm)									
≤ 5	36	81.8	0.884	65.0	0.247	92.5	0.756	68.0	0.364
> 5	78	85.7		75.9		96.0		76.8	
pT category									
T2	7	100.0	0.223	68.6	0.908	100.0	0.744	68.6	0.892
T3	96	85.5		71.6		94.2		73.4	
T4	11	60.6		85.7		100.0		85.7	
TNM stage									
IIIB	55	88.6	0.262	72.9	0.675	97.4	0.143	71.4	0.845
IIIC	59	81.2		72.2		92.3		76.8	
Lymphovascular invasion									
No	86	85.8	0.124	78.2	0.013	93.4	0.819	80.2	0.027
Yes	28	82.2		54.8		100.0		54.8	
Tumor deposits									
No	91	88.7	0.008	75.3	0.258	96.4	0.190	74.3	0.517
Yes	23	71.3		61.3		88.4		72.8	
Number of nodes retrieved									
<12	10	77.8	0.066	36.0	0.002	100.0	0.446	36.0	0.001
≥ 12	104	85.2		76.5		94.3		78.2	
Sequence									
A-CRT	71	81.8	0.379	66.7	0.153	93.7	0.629	67.5	0.074
A-CT	43	90.8		83.9		97.4		86.6	

*Abbreviations*: *OS* overall survival, *DFS* disease-free survival, *DMFS* distant metastasis-free survival, *A-CRT* primary adjuvant concurrent chemoradiotherapy, *A-CT* primary adjuvant chemotherapy

**Table 4:** Multivariate analysis of prognostic factors for 114 patients with stage N2 rectal cancer

Endpoint	Item	HR	95% CI	*p*
OS	Lymphovascular invasion (Yes vs No)	3.376	1.043-10.929	0.042
	Tumor deposits (Yes vs No)	4.604	1.516-13.976	0.007
DFS	Age (years)	0.961	0.929-0.995	0.026
	A-CT vs. A-CRT	0.345	0.137-0.868	0.024
	Lymphovascular invasion (Yes vs No)	3.324	1.520-7.269	0.003
DMFS	A-CT vs. A-CRT	0.366	0.143-0.938	0.036
	Lymphovascular invasion (Yes vs No)	2.928	1.359-6.309	0.006
	Number of nodes retrieved	0.945	0.897-0.997	0.037

*Abbreviations*: *OS* overall survival, *DFS* disease-free survival, *DMFS* distant metastasis-free survival, *A-CRT* primary adjuvant concurrent chemoradiotherapy, *A-CT* primary adjuvant chemotherapy, *HR* hazard ratio, *CI* confidence interval

## Discussion

The findings of this study demonstrate lower treatment failure and better survival (DFS and DMFS) rates when adjuvant chemotherapy was administered first in patients with pathological stage N2 rectal cancer.

With improvements in radiotherapy and surgery, regardless of whether the patient receives preoperative or postoperative chemoradiotherapy, the incidence of locoregional recurrence is relatively low; however, distant metastasis has become the predominant problem, especially in patients with stage N2 disease [3, 4]. New chemotherapy regimens have been investigated to reduce the occurrence of distant metastasis. The MOSAIC trial indicated that adding oxaliplatin to fluorouracil-based adjuvant chemotherapy significantly improved 5-year DFS and 6-year OS in stage II/III colon cancer, especially in stage III disease [13]. Although the MOSAIC trial did not include rectal cancer patients, oxaliplatin-based adjuvant chemotherapy was still recommended for rectal cancer in the National Comprehensive Cancer Network guideline [2]. Despite no head to head comparison in the adjuvant setting, current treatment guidelines accept either XELOX or FOLFOX as standard of care treatment options [2]. Randomized phase III studies indicated that XELOX is noninferior to FOLFOX as a first-line treatment for metastatic colorectal cancer [14, 15]. Most of the support for use of FOLFOX or XELOX as adjuvant chemotherapy in rectal cancer is an extrapolation from the data available for colon cancer [13, 16, 17]. The trial [18] investigating the efficacy and safety of substituting fluorouracil with capecitabine for perioperative treatment in locally advanced rectal cancer indicated that 5-year overall survival in the capecitabine group was non-inferior to that in the fluorouracil group. The authors concluded that capecitabine could replace fluorouracil in adjuvant chemoradiotherapy regimens for patients with locally advanced rectal cancer.

In a meta-analysis of the optimal interval between surgery and initiation of adjuvant chemotherapy in colorectal cancer, a 4-week increase in the time to adjuvant chemotherapy was associated with a significant decrease in both OS (HR 1.14, 95% CI, 1.10–1.17) and DFS (HR 1.14, 95% CI, 1.10–1.18) [19]. Furthermore, in the study of Kusters et al., adjuvant chemotherapy prevented local recurrence in patients with locally advanced rectal cancer [20].

So far, there have been two trials in rectal cancer evaluating treatment outcomes in relation to the sequence of adjuvant treatment [5, 6]. In a prospective randomized trial, 308 patients with resected stage II/III rectal cancer were randomly assigned to receive pelvic irradiation at either the first or the third course of leucovorin-modulated 5-fluorouracil chemotherapy [5]. In the preliminary results, a significantly higher DFS rate was achieved in the early pelvic radiotherapy group (81% vs. 70% at 4 years, *p* = 0.047) [21]. However, no significant difference in DFS, OS or relapse rate was observed between the two groups after a median follow-up period of 121 months. In the study of Kim et al., 5-year treatment outcomes were not significantly influenced by the sequence of adjuvant treatment [6].

In the present study, we focused on stage N2 patients who were more likely to develop distant metastases and local recurrence. Only patients receiving adjuvant chemotherapy (four or more cycles of XELOX or eight or more cycles of FOLFOX) were included to ensure the dose of adjuvant chemotherapy. More than 90% of patients in both groups had received a full dose of radiation, regardless of whether it was prescribed immediately after R0 resection or after adjuvant chemotherapy. With oxaliplatin adjuvant chemotherapy, the whole group exhibited high 3-year estimated OS, DFS, LRFS and DMFS rates (84.6%, 72.5%, 94.8% and 74.1%, respectively). Regarding the relationship between the timing of adjuvant radio/chemoradiotherapy and local recurrence, a systematic review indicated that the risk of local recurrence increased with waiting time for radiotherapy and the increase in local recurrence rate may translate into reduced survival in some clinical situations. However, patients with rectal cancer were not included in this review [22]. In our study, the incidence of locoregional recurrence was relatively low in both the A-CRT and the A-CT groups (5.6% and 2.3%, respectively) regardless of whether concurrent chemoradiotherapy was administered early or was delayed. However, whether adjuvant chemotherapy is given early or late does matter with regard to distant metastasis and overall recurrence. After adjusting for confounding factors with a Cox proportional hazards model, we found that giving adjuvant chemotherapy before concurrent chemoradiotherapy (A-CT vs. A-CRT) was a favorable prognostic factor for estimated DFS and DMFS. Given no significant difference in locoregional recurrence between the A-CRT group and the A-CT group in our study, we prefer to administer adjuvant chemotherapy before concurrent chemoradiotherapy after definitive surgery in pathological stage N2 rectal cancer patients.

There is a growing interest in developing neoadjuvant chemotherapy for locally advanced rectal cancer. In a phase 2, non-randomised trial of locally advanced rectal cancer, 25 (38%, 27-51) of 65 achieved a pathological complete response by adding 6 cycles of mFOLFOX6 between chemoradiation and surgery [23]. Recently, a randomized phase 3 trial indicated perioperative mFOLFOX6 alone had inferior results and a lower pCR rate than chemoradiotherapy but led to a similar downstaging rate as fluorouracil-radiotherapy, with less toxicity and fewer postoperative complications [24]. A phase 3 trial (NCT02533271) of comparing effectiveness of short-term radiotherapy plus neoadjuvant chemotherapy with preoperative long-term chemoradiotherapy in locally advanced rectal cancer is undergoing in our center.

There are several limitations to the present study, including the retrospective nature of the study design and the limited number of patients in the two groups. Furthermore, we did not determine the optimal time for intervention with radiotherapy. A further prospective randomized study is necessary for accurate evaluation of the sequence in which chemoradiotherapy should be administered after primary surgery in patients with stage N2 rectal cancer. However, conducting such a randomized controlled trial will be difficult because, since publication of the German trial, a larger number of patients with locally advanced rectal cancer have been treated with preoperative chemoradiotherapy than with postoperative chemoradiotherapy [1].

## Conclusions

In pathological stage N2 rectal cancer patients, administering adjuvant chemotherapy before chemoradiotherapy led to a lower rate of treatment failure, especially with respect to distant metastasis. Adjuvant chemotherapy prescribed as early as possible might benefit this cohort of patients in this era of oxaliplatin-based adjuvant therapy.

## Abbreviations

A-CRT: Primary adjuvant concurrent chemoradiotherapy
A-CT: Primary adjuvant chemotherapy
CI: Confidence interval
CT: Computed tomography
DFS: Disease-free survival
DMFS: Distant metastasis-free survival
FOLFOX: Fluorouracil, leucovorin, and oxaliplatin
HR: Hazard ratio
LRFS: Locoregional recurrence-free survival
MRI: Magnetic resonance imaging
OS: Overall survival
XELOX: Oxaliplatin plus capecitabine
